# Patients Selection for Immunotherapy in Solid Tumors: Overcome the Naïve Vision of a Single Biomarker

**DOI:** 10.1155/2019/9056417

**Published:** 2019-04-24

**Authors:** Diego Signorelli, Patrizia Giannatempo, Giulia Grazia, Marco Maria Aiello, Federica Bertolini, Aurora Mirabile, Sebastiano Buti, Enrico Vasile, Vieri Scotti, Pasquale Pisapia, Maria Silvia Cona, Christian Rolfo, Umberto Malapelle, Immune-Oncology YOUNG Group

**Affiliations:** ^1^Medical Oncology Department, Fondazione IRCCS Istituto Nazionale dei Tumori, Via Venezian 1, 20133, Milan, Italy; ^2^Fondazione IRCCS, Istituto Nazionale dei Tumori, Via Venezian 1, 20133, Milan, Italy; ^3^Department of Research, Human Tumors Immunobiology Unit, Fondazione IRCCS Istituto Nazionale dei Tumori, Via Venezian 1, 20133, Milan, Italy; ^4^Oncology Unit, Policlinico - Vittorio Emanuele Hospital, Via Salvatore Citelli 6, 95124, Catania, Italy; ^5^University Hospital of Modena, Via del Pozzo 71, 41124, Modena, Italy; ^6^Department of Medical Oncology, IRCCS San Raffaele Hospital, Via Olgettina 60, 20132, Milan, Italy; ^7^Medical Oncology Unit, University Hospital of Parma, Via Gramsci 14, 43126, Parma, Italy; ^8^Medical Oncology, Azienda Ospedaliero-Universitaria Pisana, Via Roma 67, 56126, Pisa, Italy; ^9^Department of Radiation Oncology, Azienda Ospedaliero Universitaria Careggi, University of Florence, Largo Brambilla 3, 50134, Florence, Italy; ^10^Department of Public Health, University of Naples “Federico II”, via Sergio Pansini 5, 80131, Naples, Italy; ^11^Marlene and Stewart Greenebaum Comprehensive Cancer Center, University of Maryland School of Medicine, Baltimore, 655 W Baltimore S, Baltimore, 21201, Maryland, USA

## Abstract

Immunotherapy, and in particular immune-checkpoints blockade therapy (ICB), represents a new pillar in cancer therapy. Antibodies targeting Cytotoxic T-Lymphocyte Antigen 4 (CTLA-4) and Programmed Death 1 (PD-1)/Programmed Death Ligand-1 (PD-L1) demonstrated a relevant clinical value in a large number of solid tumors, leading to an improvement of progression free survival and overall survival in comparison to standard chemotherapy. However, across different solid malignancies, the immune-checkpoints inhibitors efficacy is limited to a relative small number of patients and, for this reason, the identification of positive or negative predictive biomarkers represents an urgent need. Despite the expression of PD-L1 was largely investigated in various malignancies, (i.e., melanoma, head and neck malignancies, urothelial and renal carcinoma, metastatic colorectal cancer, and pancreatic cancer) as a biomarker for ICB treatment-patients selection, it showed an important, but still imperfect, role as positive predictor of response only in nonsmall cell lung cancer (NSCLC). Importantly, other tumor and/or microenvironments related characteristics are currently under clinical evaluation, in combination or in substitution of PD–L1 expression. In particular, tumor-infiltrating immune cells, gene expression analysis, mismatch- repair deficiency, and tumor mutational landscape may play a central role in predicting clinical benefits of CTLA-4 and/or PD-1/PD-L1 checkpoint inhibitors. In this review, we will focus on the clinical evaluation of emerging biomarkers and how these may improve the naïve vision of a single- feature patients-based selection.

## 1. Introduction

**Figure 1:**
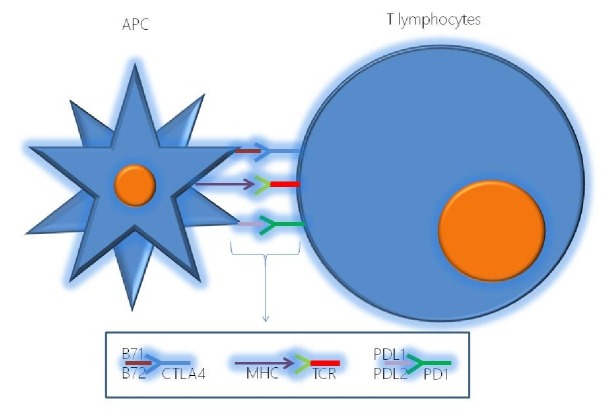
In the figure are summarized the principal immune checkpoints between the Antigen-presenting cell and T lymphocyte with a schematization of the relative molecules involved in the process (PD1 and PD-L1/PD-L2; CTLA4 and B71/B72).

Acquisition of a variable number of genetic alterations, leading to the loss of physiological cellular regulatory functions, represents one of the most important characteristics in cancer initiation and development [1, 2]. The mutations acquired by the developing cancer cells result in the expression of non-self-antigens (generally termed as neoantigens) and in the presentation of peptides bound to major histocompatibility class I (MHC-I) molecule, ultimately generating immune system activation [3]. Activated T cells can recognize cancer-specific peptide-MHC-I complex but, even when a response occurs, it rarely provides protective immunity because of the ability of tumor cells to generate an immunosuppressive microenvironment, achieving immune tolerance and immune escape mainly through the overexpression of inhibitory receptors and their ligands by immune cells and tumor cells respectively [4, 5]. Targeting the inhibition of T-cell responses using specific monoclonal antibody able to block the binding of inhibitory receptors with their ligands can lead to immune response restoration against the cancer cells [6–9]. Indeed, antibodies against CTLA-4 (i.e., Ipilimumab) or PD-1/PD-L1 (i.e., Nivolumab, Pembrolizumab, and Atezolizumab) demonstrated a relevant clinical value in different cancer patients [6–9]. However, across different solid tumors, the immune-checkpoints inhibitors efficacy is limited to a relative small number of patients and, for this reason, the identification of positive or negative predictive biomarkers represents an urgent need for a tailored therapy [10] (Figure 1).

The PD-L1 expression on tumor cells was initially identified as logical biomarker for the prediction of treatment response to anti-PD-1/anti-PD-L1 therapies, and this topic has been largely investigated across different tumor types (especially melanoma and NSCLC) with conflicting results [11]. Other tumor and/or microenvironments related characteristics are currently under evaluation, in combination or in substitution of PD-L1 expression. In particular, tumor-infiltrating immune cells, analysis of gene expression, mismatch-repair deficiency, and/or tumor mutational landscape may play an important role in predicting clinical benefits of CTLA-4 and PD-1/PD-L1 checkpoint inhibitors [12–14].

Indeed, the Food and Drug Administration (FDA, USA) has recently approved the mismatch-repair deficiency as first biomarker to positively select adult and pediatric cancer patients for pembrolizumab (PD-1 checkpoint inhibitor) treatment [15]. Conversely, in Europe further clinical trials are needed to translate the application of mismatch-repair deficiency status analysis and others putative biomarkers in clinical practice.

In the present review, we will resume the most important clinical trials for immunotherapy agents across several tumor types, focusing on the ones that evaluated the role of emerging biomarkers of response and how these results may improve the naïve vision of a single biomarker- based patients selection.

## 2. Immune-Checkpoints Inhibitors in Melanoma: The Role of Predictive Factors

Response rates of melanoma patients treated with pembrolizumab ranged around 57% in tumors with high PD-L1 expression and 8% in PD-L1 negative melanomas [16]. Initial data from CheckMate-067 trial suggested that the addition of nivolumab to ipilimumab may be more advantageous if the expression of PD-L1 was low, since PD-L1 negative patients were those who gained greater benefit from the combination, while PD-L1 positive patients had similar clinical benefits both with doublet or with monotherapy [17]. However, these preliminary findings were not confirmed in the updated results of CheckMate-067, since higher response rates for the combination have been observed regardless of PD-L1 status [18].

All these findings together suggest that melanoma patients with low/absent PD-L1 tumors do not respond to immunotherapy as well as those with high PD-L1 expression, but some PD-L1 negative patients achieve responses to anti-PD-1 antibodies becoming long-term survivors [19] and, for this reason, a low/absent PD-L1 expression does not exclude a treatment with anti-PD-1 antibodies in this tumor. On the other side, the absence of benefit in some PD-L1 positive melanoma patients implies that other molecular mechanisms are involved in resistance to check- point inhibition.

Other predictive factors for immunotherapy in melanoma are under investigation and major findings emerge from small retrospective studies. The most promising results come from tumor- infiltrating lymphocytes (TILs) and tumor mutational burden (TMB), although they are not being used in daily practice yet.

Starting from the observation that “inflamed” cancers usually respond better to immune agents than “cold” tumors, the role of TILs as predictive factor for patient's selection has been studied in melanoma and other tumor types. Importantly, a high density of TILs in tumor specimens obtained after the second dose of ipilimumab is related to higher activity of this drug [20]. Moreover, a retrospective analysis on biopsies from melanoma patients treated with pembrolizumab showed that PD-L1 positivity with a contemporary high percentage of CD8+ TILs in tumor tissue is associated with response [21]. A recent study showed that melanoma patients treated with anti-PD-1 antibodies presented an objective response rate (ORR) of 79% when baseline melanoma specimens had more than 20% of PD-1^high^/CTLA-4^high^ CD8+ TILs while patients with fewer than 20% of PD- 1^high^/CTLA-4^high^ CD8+ TILs were nonresponders [22]. Indeed, it has recently been observed that increase of memory CD8+ TILs between baseline and posttreatment biopsies after a therapy with anti-PD-1 antibodies is associated with clinical benefit [23].

Best responses to immune agents were seen in melanomas and NSCLCs, both widely related to chronic exposure to mutagens (ultraviolet light and smoke's carcinogens, respectively). It was hypothesized that tumors with a large number of somatic gene mutations develop a more elevated anti-PD1-induced neoantigen-specific T-cell response which results in an increased susceptibility to immunotherapy [24, 25]. For this reason, the TMB represents a novel candidate as biomarker of response to immune agents. Two studies explored the correlation between higher TMB (assessed by whole-exome sequencing technique) and benefit from anti-CTLA-4 antibodies in melanoma patients. They found that a TMB higher than 100 somatic mutations is correlated with increased responses and overall survival (OS) [26, 27]. The mutational load is a very promising biomarker limited by cost barriers and informatics requirements correlated to the whole-exome sequencing assay. A recent retrospective analysis suggests the possibility of using a small set of genes as alternative to the whole-exome to predict response to ipilimumab [28].

Moreover,* BRAF*-mutant melanomas are considered more aggressive than wild-type tumors, but the role of this genetic alteration as predictive biomarker for immunotherapy is debated. In a retrospective analysis of melanoma patients treated with pembrolizumab in second-line, the ORR was 26% for wild-type melanomas and 12% for* BRAF*-mutant patients [29]. A similar difference, even if smaller, was observed in the first-line setting with pembrolizumab: the rates of response were 38% for wild-type patients and 32% for* BRAF*-mutant subjects. Conversely, the CheckMate- 067 trial with nivolumab showed that* BRAF*-mutant patients achieved a higher 2-year survival rate than wild-type melanomas (62% versus. 57%, respectively) [30]. Based on these data, today* BRAF* status is not considered a predictive factor for melanoma patients receiving immunotherapy.

Peripheral blood markers are far from being accurate and specific biomarkers, but have the advantage of being more easily and quickly used by oncologists in everyday clinical practice than molecular analyses. Higher lactate dehydrogenase (LDH) levels are frequently related to lower rates of response to immunotherapy in melanoma trials: in the first-line setting, the ORR for pembrolizumab was 40% for patients with normal levels of LDH, 34% for subjects with an elevation of LDH up to twice the upper normal limit (UNL), and 8% for patients with LDH increased over twice the UNL [9]; similarly, the ORR of pembrolizumab in second-line was 26% for subjects with higher LDH levels and 34% for the total population [31]. Response to immune-checkpoints antibodies also seems to correlate with some pretreatment white blood cells elements: a lower neutrophil count, a lower neutrophil to lymphocyte ratio (NLR), and a higher eosinophil count are associated with clinical benefit in advanced melanoma patients treated with ipilimumab [32, 33]. Probably, these peripheral blood factors do not directly affect response to immunotherapy, but may reflect the achievement of molecular mechanisms of resistance to checkpoints inhibitors by melanoma cancer cells.

## 3. The Role of Predictive Factors to Immune-Checkpoints Inhibitors in NSCLC

Similarly to melanoma, the role of PD-L1 expression and TMB as possible biomarkers for response to ICB was investigated also in NSCLC.

The phase III KEYNOTE-024 trial showed pembrolizumab as new standard first-line treatment in advanced NSCLC with high PD-L1 expression, defined as expression in at least 50% of tumor cells, tumor proportion score (TPS) ≥50%, and absence of* EGFR* or* ALK* aberrations [6]. In comparison with standard platinum-based chemotherapy, pembrolizumab improved progression free survival (PFS), the primary endpoint (10.3 versus. 6.0 months, hazard ratio (HR): 0.50; 95%CI 0.37-0.68; p<0.001), OS (6 months OS rate 80.2% versus. 72.4%; HR: 0.60, p=0.005) and response rate (RR) (44.8% versus. 27.8%) [6]. In the KEYNOTE-042 (NCT02220894), pembrolizumab was compared with carboplatin-based chemotherapy in patients with lower PD-L1 expression (TPS≥1%). The trial met its primary endpoint, OS [34]. Differently from these results, in the phase III CheckMate-026 trial, monotherapy with nivolumab did not show higher efficacy than platinum-based chemotherapy in patients with PD-L1 expression greater than 5%; also in the exploratory subgroup analysis in patients with PD-L1 expression ≥ 50%, nivolumab was not associated with better progression free survival (PFS), OS or RR. Another exploratory analysis showed that high TMB, defined as the highest thirds of somatic missense mutations (243 or more) in the baseline evaluable tumor samples, was related to better RR and PFS with immunotherapy than chemotherapy (47% versus. 28% and 9.7 versus. 5.8 months, respectively); probably because of high treatment crossover (68% of patients with high TMB in chemotherapy arm received nivolumab afterwards), no difference in OS was detected. Importantly, there was no correlation between TMB and PD-L1 expression level [39].

To date, different clinical trials are focused on strategy combinations in first-line setting.

The double-blind phase III randomized placebo-controlled KEYNOTE-407 and KEYNOTE-189 trials evaluated the combination of pembrolizumab with platinum-based chemotherapy in advanced squamous NSCLC and nonsquamous NSCLC without* EGFR* or* ALK* alterations, respectively. Patients with any level of PD-L1 expression were included. The trials showed a benefit for pembrolizumab combination arms in both the primary endpoints, OS and PFS; the benefit was evident across all PD-L1 categories (TPS <1%, 1-49%, ≥50%) [37, 38]. In the open-label phase III IMpower 131 and IMpower 150 trials, including patients with squamous and nonsquamous advanced NSCLC respectively and any PD-L1 expression, the combinations of atezolizumab plus carboplatin and nab-paclitaxel (IMpower 131) or plus carboplatin, paclitaxel and bevacizumab (IMpower 150) were related to better PFS than chemotherapy or chemotherapy plus bevacizumab alone. If in the squamous histology PFS benefit was more appreciable in PD-L1 positive patients, especially in those with high expression (TC3 or IC3), in IMpower 150 the benefit was evident regardless of PD-L1 status, including PD-L1 negative patients. The first interim OS results were positive in the nonsquamous histology while in IMpower 131 the benefit was detectable only in patients with high PD-L1 expression [47, 48]. The open-label phase III IMpower 132 randomized nonsquamous NSCLC patients, without* EGFR* or* ALK* aberrations, to receive cisplatin/carboplatin plus pemetrexed and atezolizumab or chemotherapy alone. The trial showed an improvement in PFS for the experimental arm, while an interim analysis did not find a statistical significant benefit in OS, in spite of a difference of 4.5 months. In an exploratory analysis, patients with high or negative PD-L1 expression achieved the most benefit from immunotherapy [49].

Different studies have analyzed the double immune-checkpoints blockade. In the phase I CheckMate-012 trial, despite having more toxicity, the combination of nivolumab plus ipilimumab showed higher efficacy than nivolumab alone (ORR 43% versus 23%). To note, the combination increased more than twice the ORR in patients who are low responders to immunotherapy, such as never smokers (ORR 27% versus. 9%) and* EGFR*-mutated patients (ORR 50% versus. 14%). The combination was associated with longer PFS (median PFS: 8.0 versus. 3.6 months) and higher 1-year OS rate (76% versus. 73%) among patients unselected for PD-L1 expression, although the efficacy increased with increasing PD-L1 expression (RR 92% in patients with PD-L1 expression ≥50%) [78]. The open-label, multipart phase III CheckMate-227 trial evaluated the role of nivolumab-based regimens in advanced NSCLC patients without* EGFR* mutations or* ALK* translocations. Compared to chemotherapy alone, nivolumab plus ipilimumab improved PFS in patients with high TMB (amended coprimary endpoint), defined as at least 10 mutations per magabase (7.2 versus 5.5 months, HR 0.58, 97.5%CI 0.41-0.81, p<0.001). PFS was longer in the combination arm regardless of PD- L1 expression level; again, no significant association was detected between TMB and PD-L1 expression. Regarding nivolumab monotherapy, in patients with high TMB and PD-L1 expression ≥1%, no benefit in PFS was achieved as compared to standard chemotherapy. Therefore, the trial validated the role of TMB as PD-L1 independent positive predictive biomarker in advanced NSCLC patients treated with nivolumab plus ipilimumab. However, only about 58% of patients showed adequate data for TMB-based efficacy analysis [14].

Several other studies investigated the activity of ICB in second or further lines of therapy for NSCLC and the role of PD-L1 expression or TMB; all the published randomized trials had docetaxel as control arm and OS as primary endpoint.

The first published phase III studies were conducted with nivolumab in squamous (CheckMate-017) or nonsquamous (CheckMate-057) advanced NSCLC [40, 41]. Both studies achieved their primary endpoint: median OS was 9.2 versus. 6 months, HR 0.59, 95%CI 0.44-0.79, p<0.001, in CheckMate-017 [40]; 12.2 versus. 9.4 months, HR 0.76, 96% CI 0.59-0.89, p=0.002, in CheckMate-057 [41]. PD-L1 expression was retrospectively evaluated on available archival or fresh tissue samples (83% and 78% of patients in CheckMate 017 and 057, respectively). In CheckMate- 017, PD-L1 expression did not show any prognostic or predictive role while in 057 its higher percentage (≥1%, ≥5%, ≥10%) was related to better OS, PFS, and ORR, even if also PD-L1 negative patients achieved benefit. The pooled analysis on PD-L1 showed a relationship between increasing expression and better OS [42].

Based on phase I study data (KEYNOTE-001), which showed more efficacy of pembrolizumab with higher PD-L1 expression and 50% as cut off related to an increase in ORR, PFS, and OS [35], a randomized phase II/III trial was performed on 1034 patients, with PD-L1 at least on 1% of tumor cells as inclusion criterion (KEYNOTE-010). The trial showed improved OS with immunotherapy and the benefit was greater in patients with PD-L1 expression ≥50% [36].

The randomized phase II POPLAR and the phase III OAK studies showed a benefit of the anti-PD-L1 agent atezolizumab in both OS and safety [43, 44]. The single arm phase II BIRCH study enrolled only PD-L1 positive patients and showed survival data similar to those of POPLAR and OAK [45]. Differently from trials with other immune-checkpoints inhibitors, in these three studies PD-L1 expression was separately assessed on both tumor cells (TC) and tumor-infiltrating immune cells (IC). Even if efficacy was greater in patients with higher PD-L1 expression, OAK study showed an improved survival with atezolizumab also in the PD-L1 low or undetectable subgroup (TC0 and IC0).

For what concerns other possible predictive biomarkers, high expression on tumor specimens of T- effector and interferon-*γ* gene signature (defined by CD8A, GZMA, GZMB, IFN*γ*, EOMES, CXCL9, CXCL10, and TBX21) was related to improved OS in POPLAR study [43]. Furthermore, a 394 gene-based next generation sequencing (NGS) assay was used to retrospectively test plasma samples for blood tumor mutational burden (bTMB) from POPLAR and OAK. The cut-point of bTMB ≥16 was selected in POPLAR, and independently validated to predict PFS benefit in OAK. As already shown on tissue samples in CheckMate 026 and 227 [14, 39], also bTMB was not significantly associated with tumor PD-L1 expression [46].

Durvalumab was tested in a phase II, open-label, single-arm trial (ATLANTIC) in patients with stage IIIB–IV NSCLC, that progressed to at least 2 prior systemic treatment regimens. The study considered three cohorts; the first one included* EGFR*-mutant/*ALK*-positive patients, the second and the third cohorts the* EGFR*/*ALK* wild-type patients with different PD-L1 expression (low/negative or ≥25% in cohort 2, ≥90% in cohort 3). Confirmed responses per independent central review in* EGFR*/*ALK *wild-type patients were 7.5% (95%CI 3.1-14.9) in PD-L1 low (<25%)/negative patients, 16.4% (95%CI 10.8-23.5) in patients with PD-L1 expression ≥25%, 30.9% (95%CI 20.2-43.3) in cohort 3 (PDL-1≥90%). Interestingly, the rate of immune-mediated adverse events was twice as high in cohort 3 in comparison with cohort 2 [50]. In a phase Ib study, durvalumab was tested in combination with the CTLA-4 inhibitor tremelimumab. Most of the patients (96/102) had already undergone at least one prior line of systemic therapy. In the combined T1 cohort (durvalumab 10–20 mg/kg plus tremelimumab 1 mg/kg), the combination was effective regardless of PD-L1 expression; ORR was 22%(95% CI, 3–60) in patients with PD-L1 positive tumors (≥25% of tumor cells with membrane staining for PD-L1) and 29% (95% CI, 8–58) in PD-L1 negative patients, including patients with no PD-L1 staining [52].

In a cohort of a phase I study in pretreated patients, avelumab, in most of the cases (66%) the second line of systemic therapy, showed an ORR of 12% and 1-year OS rate of 36%. According to a 1% threshold, 122 (86%) of 142 evaluable tumor samples were PD-L1 positive. ORR and overall survival did not significantly differ between patients with PD-L1 positive and PD-L1 negative tumors [53]. A phase III study comparing avelumab versus docetaxel as second-line therapy (JAVELIN Lung 200 trial) did not meet OS in PD-L1 positive (≥1% of tumor cells) population, the primary endpoint; PD-L1 expression and NSCLC histology were stratification criteria [54].

If immunotherapy has clearly improved outcome of patients with* EGFR*/*ALK* wild-type NSCLC, the benefit in subjects with mutated* EGFR *or rearranged* ALK* is not clear. Except for IMpower 150,* EGFR* and* ALK *alterations were exclusion criteria for the first-line phase III trials above. A single center phase II trial with pembrolizumab in tyrosines kinase inhibitors (TKIs) naïve patients with* EGFR* mutations (sensitizing or not) and PD-L1 expression ≥1% was prematurely stopped for futility. In spite of the high proportion (70%) of PD-L1 strong positive (≥50%) tumors, none of the 10 patients with documented* EGFR* mutations achieved an objective response [79]. Considering the second or further lines, in a meta-analysis on three randomized trials (CheckMate-057, KEYNOTE-010, POPLAR) and 1548 patients with known* EGFR *mutation status, immune-checkpoints inhibitors did not improve OS in the* EGFR*-mutant subgroup (N= 186) compared to docetaxel (HR 1.05, IC 95%: 0.7-1.55, p = 0.81; heterogeneity p= 0.80) [80]. The subgroup analysis from 85 patients with* EGFR *mutations in OAK study showed a similar result (HR 1.24, 95%CI 0.71-2.18) [43]. No responses were detected among the patients with mutated* EGFR *(N= 9) or* ALK *translocation (N=1) in the phase I study with avelumab [53]. In the only trial that prospectively evaluated immunotherapy in* EGFR*-mutant/*ALK*-positive patients with a specific cohort (ATLANTIC), ORR was 12.2% (95%CI 5.7-21.8) in high (≥25%) PDL-1 expression subgroup, only 3.6% (95%CI 0.1- 18.3) in patients with PDL-1 low (<25%) or negative [50]. Importantly, the poor efficacy of immunotherapy in this population can be related to the low TMB [81]. Combinations of immune-checkpoints inhibitors with TKIs have been characterized by very high toxicities, with consequent modification of the therapeutic schedule (i.e., nivolumab plus ceritinib) [82] or even permanent interruption of the trial (i.e., durvalumab plus osimertinib in TATTON study) [83]. Based on these data, it is still controversial the possible role of immunotherapy in patients with mutated* EGFR *or rearranged* ALK*.

Considering the achieved benefit in advanced disease, several clinical trials are evaluating the role of immunotherapy in early stages in NSCLC. In a pilot study, nivolumab was administered up to two doses as neoadjuvant treatment in 21 patients with stage II or IIIA disease. The trial met its primary endpoint (safety and feasibility), with no treatment-related surgical delays. Despite only 2 (10%) patients achieved a partial response according to RECIST 1.1 criteria, a major pathological response (MPR, defined as 10% or less of residual viable tumor cells in the resected primary tumors) was detected in 9 (45%) of the 20 patients who underwent complete tumor removal. MPR occurred in both PD-L1 positive and negative tumors while it was significant associated with higher TMB. Tumors with MPR had a higher clonality of T-cell population than tumors without MPR [84]. Immunotherapy changed the clinical practice also in the particular setting of locally advanced, unresectable stage III disease. Based on preclinical data of upregulation of PD-L1 expression on tumor cells after chemotherapy and radiotherapy [85], in the double-blind phase III PACIFIC trial, durvalumab up to 1 year improved median PFS over placebo in patients with stage III A/B after concomitant definitive chemoradiation (16.8 versus 5.6 months, HR 0.52, 95%CI 0.42-0.65, p<0.001), with an acceptable toxicity profile. A longer PFS was achieved regardless of PD-L1 expression and also in patients who do not generally benefit from immunotherapy, as never smokers [86]. Durvalumab prolonged also OS than placebo; a post hoc exploratory analysis did not show a significant OS benefit in patients with PD-L1 expression <1% [51].

## 4. Patients Selection for Immunotherapy: The Role of Genetic Instability in Metastatic Colorectal Cancer Patients

Microsatellite instability (MSI) is a molecular marker of a deficient mismatch repair (dMMR) system and occurs in approximately 15% of colorectal cancers (CRCs). “High” microsatellite instability (MSI-H) CRCs has distinct clinical and pathological features such as proximal location, early stage (predominantly stage II), poor differentiation, mucinous histology, and association with* BRAF* mutations [87]. In early-stage CRC, MSI identifies a group of tumors with a better prognosis, while in metastatic disease it seems to confer a negative prognosis. While the dMMR phenotype remains a favorable prognostic factor in patients with stage III CRCs receiving FOLFOX adjuvant chemotherapy [88], patients with dMMR metastatic CRCs and MSI-H have a poor prognosis due to low efficacy of traditional chemotherapy related to high mutational burden, tumor neoantigen load, and infiltration of immune cells. This may justify an endogenous immune antitumor response, counterbalanced by the expression of immune inhibitory signals, such as PD-1 or PD-L1. Based on these considerations, MSI-H CRCs seem to be particularly responsive to immunotherapy, such as anti-PD-1 [12]. Different recent published reports demonstrated the role of anti-PD-1 immune-checkpoints inhibitors in patients with progressive metastatic CRCs with dMMR. In 2015 Le DT et al. firstly published data on 41 patients with MMR deficient and proficient (pMMR) CRCs treated with pembrolizumab in a phase 2 study. The study reached the primary endpoint, showing an immune-related and RECIST ORR of 40% (4 of 10 patients), a disease control rate (DCR) of 90% and an immune-related PFS rate at 20 weeks of 78% (7 of 9 patients) in the cohort of dMMR CRCs. On the other hand, in the cohort of pMMR CRCs no immune-related and RECIST ORR were observed and the immune-related PFS rate at 20 weeks was 11% [25]. Based on these considerations, a phase II (KEYNOTE-164) and a phase III (KEYNOTE-177) clinical trials evaluating pembrolizumab in dMMR CRCs are ongoing [7, 8]. Overman et al. published a phase 2 trial on 74 patients with dMMR/MSI-H heavily pretreated recurrent or metastatic CRCs treated with nivolumab. Primary endpoint was RECIST ORR: 23 of 74 patients (31%) achieved an objective response (all partial responses) and 51 (69%) patients had disease control for 12 weeks or longer. Eight patients had responses lasting 12 months or longer. Responses were durable; median duration of response had not yet been reached and the median progression free survival was 14.3 months. Tumor expression of PD-L1 was not predictive of response. Taken together, the results of this phase II trial suggest that nivolumab monotherapy has durable antitumor activity in patients with dMMR/MSI-H colorectal cancer, supporting further investigation of nivolumab alone or in combination with other therapies [55]. Moreover, promising preliminary analysis of the combo ipilimumab and nivolumab in patients with dMMR/MSI-H was presented at ASCO 2017 [56]. Twenty-seven patients were treated; median time to response was 2.7 months and 82% of responses (9/11) were ongoing at 6 months. Median duration of response, PFS and OS had not been reached. CheckMate-142 is still ongoing [55].

## 5. Immunotherapy for Head and Neck Cancer: “Another Break in the Wall”

In 2016, ICB therapy demonstrated to be an important therapeutic option also in HNSCC, with favorable results in second-line clinical trials and obtained FDA approval [89]. The rational of immunotherapy is that HNSCC has immunosuppressive traits caused by several mechanisms as the impairment of tumor-infiltrating T lymphocytes and of natural killer (NK)–cell activity, the poor antigen-presenting function, accumulation of tumor-secreted proteins that inhibit stimuli, T-cell apoptosis and the presence of T-regulatory (Treg) cells that repress T cells induction and proliferation [90]. The development and progression are facilitated by the acquisition of the capacity to evade immune surveillance and an effective immune response, which is mediated in part by expression of the programmed death ligands (PD-L1 and PD-L2) of the T-cell–suppressive immune-checkpoint receptor programmed death 1 (PD-1) with a strong impact in clinical outcome [90]. The second-line phase III trial CheckMate-141 randomized recurrent and/or metastatic platinum-resistant/refractory HNSCC patients to receive either single-agent nivolumab or standard monotherapy (methotrexate, docetaxel, or cetuximab). Nivolumab improved the ORR (13% versus 6%) with long-lasting tumor regressions and median OS (7.5 versus 5.1 months, p = 0.01, hazard ratio for death 0.7), regardless of tumor PD-L1 expression or p16 status [57]. In the nonrandomized, multicohort phase Ib study KEYNOTE-012, treatment with pembrolizumab showed similar results, even if they need to be confirmed by phase III trials actually ongoing [58]. Based on these evidences, nivolumab and pembrolizumab have been approved by FDA as the new standard-of-care options for the second-line treatment of recurrent and/or metastatic HNSCC. Nevertheless, the importance of PDL-1 as biomarker is still controversial in HNSCC patients.

Mattox et al. demonstrated that in relapsed or metastatic HNSCC, it did not correlate with OS and PFS [91]. Some doubts emerged also from the randomized CheckMate-141 trial, where the survival benefit was greater in PD-L1 ≥1% population, hazard ratio for death 0.55 (0.36 to 0.86) versus. 0.89 (0.54 to 1.85), but not for increasing PD-L1 expression [57].

Considering the combined positivity score (CPS: ≥1% of expression in both tumor and mononuclear inflammatory cells), in KEYNOTE-012 trial PD-L1 positivity was related to better RR, PFS, and OS, than considering only the tumor proportion score (≥1% of expression only in tumor cells) [58]. Unfortunately, KEYNOTE-055 study did not confirm these results since the PD- L1 positivity was not predictive of response [59].

PD-L2 and tumor-infiltrating cells characterization or PD-L1 evaluation in circulating cells (blood biopsy) could be other interesting biomarkers.

In fact, Yearley et al. [60] showed a greater response in PD-L1 and PD-L2 positive patients (27.5%) than those positive only for PD-L1 (11.4%), with longer median PFS and OS for PD-L2-positive than for PD-L2-negative patients. Moreover, PD-L2 status revealed as a significant predictor of PFS with pembrolizumab, independent of PD-L1 status.

Therefore, PD-L1 is actually “a marker”, but in the development of immune response are involved so many factors so that a better knowledge is still required to define “robust and consistent biomarkers”.

## 6. Immunotherapy for Metastatic Clear Cell Renal Cell Carcinoma: How to Select the Right Patient

In metastatic renal cell carcinoma (mRCC) immune system plays a fundamental role: proofs include spontaneous remissions of metastases, positive impact on survival of nephrectomy followed by interferon-*α* (IFN-*α*) immunotherapy versus only IFN-*α* and the clinical demonstration of the antitumor activity of cytokines interleukin-2 (IL-2) and IFN-*α*, alone or in combination [92]. High dose bolus of IL-2 is currently the only cytokine monotherapy still included in the international guidelines as first-line option for highly selected clear cell mRCC patients with excellent performance status (PS) and normal organ function. The second- and third-line therapy of mRCC was recently revolutionized by the introduction of two new drugs, cabozantinib and nivolumab. They demonstrated to prolong OS compared with the (now old) standard of therapy everolimus in two phase III pivotal studies, the METEOR and the CheckMate-025 trials respectively [61, 93, 94].

A meta-analysis of six published trials showed that higher levels of tumor PD-L1 expression increase the risk of death (HR 0.81 95%CI 1.31-2.49,* p* < 0.001), defining it as a negative prognostic factor [95]. Unfortunately, the OS benefit obtained with nivolumab in the CheckMate- 025 trial was independent of PD-L1 tumor expression [61], with the limit that it was not a stratification factor for randomization in this study. However, these data are recently challenged by the results of the CheckMate-214 study, a phase III randomized open-label trial of nivolumab/ipilimumab combination versus sunitinib in first-line mRCC (1096 patients enrolled) [62]. Seventy-four percent of patients in the combination immunotherapy arm and 71% treated with sunitinib had negative (< 1%) PD-L1 tumor expression, while 26% and 29%, respectively, were PD-L1 positive (≥ 1%). The coprimary endpoint included overall RR, PFS, and OS for intermediate- and poor-risk patients (according to the International Metastatic Renal-Cell Carcinoma Database Consortium model) [96]. The study was positive in this population, with statistically significant and clinically relevant OS and ORR improvement in favor of nivolumab/ipilimumab treatment. Interestingly, PD-L1 expression, an exploratory endpoint in this study, was predictive of both RR and PFS but not of OS [62]. These results seem to be in contrast with second-line data for nivolumab, but it is important to note that a biomarker obtained from primary tumor immediately before first-line treatment is not affected by the effect of first-line treatment on tumor tissue. Moreover, in a recent study, the high c-MET expression was significantly associated with PD-L1 overexpression (*p* = 0.001), in patients with mRCC treated with first-line sunitinib, suggesting a potential benefit from combining cabozantinib and nivolumab [97]. Moreover, also a hypermutational status of the tumor has been reported as a possible predictive marker favoring the response probability to the PD-1/PD-L1 axis blockade [24].

## 7. Immunotherapy in Upper Gastrointestinal Tract Cancer

In September 2017, FDA approved pembrolizumab for previously treated patients with recurrent locally advanced or metastatic gastric or gastroesophageal junction cancer whose tumors express PD-L1, and nivolumab for the treatment of hepatocellular carcinoma patients previously treated with sorafenib [98, 99]. In metastatic gastric cancer, pembrolizumab granted approval on the basis of KEYNOTE-059 study, an open-label, multicenter, multicohort trial that enrolled 259 pretreated patients with gastric or gastroesophageal junction adenocarcinoma [63, 98]. Patients had progressed to at least 2 previous lines of treatment, so about half of patients received pembrolizumab as third-line treatment and half as fourth-line. Fifty-five percent of patients (n=143) had tumors expressing PD-L1, with PD-L1 positivity based on at least 1% of positive staining cells (tumor or stromal cells). Response rate for these 143 patients was 15.5% (95% CI: 10.1, 22.4) with 2% complete and 13.5% partial responses and with duration of response ranged from 2.8+ to 19.4+ months, with 11 patients (58%) having response durations of 6 months or longer and 5 patients (26%) having response durations of 12 months or longer. A phase III trial compared nivolumab versus placebo as third or further line of treatment in 493 Asian patients with advanced gastric cancer [64]. OS was prolonged with nivolumab with HR of 0.63 (95% CI 0.51-0.78) and a benefit in estimated survival at 12 and 18 months from 10.9% to 26.2% and from 5% to 16.2%, respectively. The study had no biological criteria of selection but a subgroup exploratory analysis on PD-L1 positivity showed an OS benefit in both positive (PD-L1≥1%; HR 0.51; 95% CI 0·21– 1·25) and negative (PD-L1<1%; HR 0·72; 95% CI 0·49–1·05) tumors. For hepatocellular carcinoma, FDA granted accelerated approval of nivolumab for patients progressed or intolerant to sorafenib. Approval was based on the results of the CheckMate-040 trial, a phase I-II study including three groups of patients (without viral hepatitis, HCV infected and HBV infected) with a dose escalation and an expansion cohort, which enrolled 262 patients with Child-Pugh A cirrhosis [65, 99]. An objective response was observed in 42 patients (20%; 95% CI 15–26) who received nivolumab 3 mg/kg every 2 weeks in the dose-expansion phase with 3 complete and 39 partial responses. Stable disease was observed in 96 (45%) patients, with 64% as DCR. The median duration of response was 9.9 months (95% CI 8.3 to not estimable). The 6-month OS rate was 83% (95% CI 78–88) and the 9-month OS rate was 74% (95% CI 67–79). Patients with PD-L1 positive tumors (defined as ≥1%) resulted only 20% of all enrolled patients; RR was similar among positive and negative patients (26 versus 19%) and FDA approved nivolumab regardless of PD-L1 positivity.

As for colorectal cancer and other malignancies, also genetic instability and mutational burden may play a major role on sensitivity of upper gastrointestinal tumors to immunotherapy. The approval of pembrolizumab in MSI-high tumors was based on the results of KEYNOTE 158 and 164 studies including both colorectal and noncolorectal cancers; the most common noncolorectal tumor types included upper gastrointestinal cancers as small intestinal cancer, cholangiocarcinoma, and gastric and pancreatic cancer [66]. Among the 259 patients enrolled in KEYNOTE-059, 7 (3%) had tumors that were determined to be MSI-high. Responses were observed in 4 of these 7 patients (57%), with one complete response and duration ranged from 5.3+ to 14.1+ months. Both PD-L1 positivity and MSI-high status contributed to better select patients who benefited from pembrolizumab, but excluded also a group of responding patients; therefore, the selection process should be implemented. However, MSI-high is a rare condition in upper gastrointestinal cancers and may be too much restrictive for the selection of sensitive patients, selecting only a small proportion (about 15%) of patients with high TMB [100].

## 8. Immunotherapy in Breast Cancer: The Last but Not the Least

Breast cancer has been classically considered poorly immunogenic. Clinical trials aimed to target immune system have relatively recently started, firstly in the metastatic setting, and the first significant results from them are now available. Among breast cancer subtypes, triple-negative breast cancer (TNBC) is characterized by greater stromal and intratumoral TILs, higher PD-L1 expression and TMB, features that make it a particularly suitable candidate for immunotherapy [101]. The phase II KEYNOTE-086 trial investigated pembrolizumab in metastatic TNBC, with 170 pretreated patients in cohort A, and 84 PD-L1 positive treatment-naïve patients in cohort B. In cohort A, median PFS and OS were 2 months (95% CI, 1.9-2.0) and 9 months (95% CI, 7.7-11.2); ORR was 5.3% (95% CI, 2.7-9.9) overall and 5.7% (95% CI, 2.4-12.2) in the PD-L1 positive populations, DCR was 7.6% (95% CI, 4.4-12.7) and 9.5% (95% CI, 5.1-16.8), respectively [67]. In cohort B, median PFS and OS were 2.1 months (95% CI, 2.0-2.2) and 18 months (95% CI, 12.9-23.0); ORR, DCR, and median duration of response were 21.4% (95% CI, 13.9-31.4), 23.8% (95% CI, 15.9-34.0), and 10.4 months (4.2-19.2), respectively [68]. The increased response in cohort B may be related to the use of pembrolizumab in a selected population of previously untreated patients with PD-L1 positive tumors. Median TILs levels were higher in cohort B (17.5% versus 5% in cohort A), and a significant correlation between PD-L1 expression and TILs levels was found (*ρ* = 0.4962, p < 0.001) [69]. Pembrolizumab has been tested also in HER-2 and in hormone receptor positive metastatic breast cancer (MBC). In the phase Ib/II trial PANACEA, pembrolizumab plus trastuzumab appeared to be well-tolerated and showed clinical benefit in HER- 2/PD-L1 positive MBCs that were trastuzumab resistant. If in the PD-L1 negative cohort no objective response was observed, in the PD-L1 positive cohort ORR and DCR were 15.2% and 24%, respectively, with 11.1 months as median duration of response. Furthermore, stromal TILs were identified as potential predictive marker: in the subgroup of patients with at least 5% of TILs in the metastatic lesion, ORR increased to 39% (versus 5% in patients with TILs levels <5%) [70]. In the multicohort phase Ib KEYNOTE-028 trial, pembrolizumab was investigated in 25 previously treated, PD-L1 positive, Estrogen Receptor positive/HER2 negative MBCs; ORR was 12% (95% CI, 2.5%-31.2%) with a median duration of response of 12 months (range: 7.4-15.9 months) [71]. A subsequent biomarkers analyses across all the cohorts showed that PD-L1, T-cell–inflamed gene-expression profile and TMB, separately or in combination, could be used to predict clinical benefit from pembrolizumab, regardless of tumor type [72].

Avelumab was administered as single agent in a cohort of the phase Ib JAVELIN trial in a heavily pretreated population. Among the 168 enrolled patients, 15.5% were HER-2 positive, 42.9% were hormone-receptor-positive/HER-2-negative and 34.5% were TNBC. Antitumor activity was modest in the overall population, with 3% as confirmed ORR, which improved in patients with PD-L1 positive tumor-associated immune cells (16.7% versus 1.6% in PD-L1 negative) and in TNBC (22.2%) [74].

Based on the results from single-agent ICB, studies of combination with chemotherapy were developed; most of them are still ongoing. IMpassion-130 is the first phase III study which demonstrated a significant benefit of immunotherapy combined to chemotherapy in metastatic TNBC in the first-line setting. Patients were randomized to receive nab-paclitaxel plus atezolizumab or placebo. In the ITT population median PFS was 7.2 months with atezolizumab and 5.5 months with chemotherapy alone (HR: 0.80, p: 0.002). A greater benefit was observed in the PD-L1 positive subgroup (median PFS 7.5 versus 5.0 months, HR 0.62, p < 0.0001). At the last interim analysis, after a median follow-up of 12.9 months, OS was 21.3 months in the experimental arm and 17.6 months in the control group, with a meaningful improvement in PD-L1 positive population (25 versus 15.5 months, respectively) [75]. PD-L1 expression resulted in the most robust predictive biomarker of PFS and OS benefit in atezolizumab treated patients. Differently from stromal TILs, intratumoral CD8 cells well correlated with PD-L1 expression and were predictive of both PFS and OS [76].

Several clinical trials are ongoing in early stages breast cancer, with the first results coming from the neoadjuvant setting. Promising data arose from the phase II I-SPY 2 trial, with pembrolizumab plus standard chemotherapy in high-risk, HER2-negative breast cancer patients. In comparison with chemotherapy alone, the addition of pembrolizumab improved pathological complete response (pCR) rates, especially in TNBC (71.4% versus 19.3% in control arm) [102]. In a small cohort of TNBC patients in the phase Ib KEYNOTE-173 study, the combination of pembrolizumab plus conventional neoadjuvant chemotherapy achieved high pCR rates (up to 100% in a cohort with the addiction of carboplatin) [103]. High levels of stromal TILs and PD-L1 expression were significantly associated with pCR and ORR [73]. These findings support the ongoing phase III KEYNOTE-522 trial. In the randomized phase II GeparNuevo study durvalumab or placebo was added to standard neoadjuvant chemotherapy in patients with early-stage TNBC. PCR rate was higher in the durvalumab arm (53.4% versus 44.2%), even if not statistically significant (p: 0.287). Interestingly, efficacy improved when durvalumab was administered for a window of 2 weeks before chemotherapy, triggering the immune system first: pCR rates moved to 61% versus 41.4% (p: 0.052) in the two arms, respectively. Patients with any level of TILs expression achieved a benefit from durvalumab addition, especially patients with the highest levels [77]. These early data are very encouraging, particularly in a poor prognosis subtype like TNBC.

## 9. Conclusion

Today, in Europe, PD-L1 expression evaluation on tumor cells by IHC with Tumor Proportional Score (TPS) is the only immune-checkpoint inhibitor positive predictive biomarker approved for NSCLC patients in first- and second-line treatment, in particular for pembrolizumab [6, 36]. Unfortunately, IHC for PD-L1 has not showed a transversal and definitive role to predict the immune-checkpoint inhibitor response in other cancers or in different settings. In addition, in many clinical trials some PD-L1 negative patients also reveal the response to PD-1/PD-L1 checkpoint inhibitor. Moreover, even though the technical issues relative to the implementation in clinical practice of PD-L1 expression evaluation (such as different antibodies and different platforms) have been exceeded, the IHC expression evaluation at a single time point may not fully represent the dynamic and complex evolution of microenvironment and tumor cells communication network [104]. Circulating miRNA signature classifier with prognostic value could integrate PD-L1 expression to identify patients with worse outcome [105]. Despite these limitations, other very interesting biomarkers were evaluated as predictors of ICB treatment response [12–14]. Among the most studied, an important role in predicting clinical benefits has been played by tumor microenvironment and, in particular, by tumor-infiltrating immune cells [12–14]. Unfortunately a quantitative and repeatable evaluation is difficult to achieve on routine histological and cytological samples, affecting a robust evaluation in different clinical trials and the introduction in clinical setting.

Recently, IHC for mismatch-repair status protein evaluation and DNA-based microsatellite instability assessment has shown an important role in immune-checkpoint inhibitor clinical efficacy prediction in different settings, for both adult and pediatric cancers, so that FDA approved its use as the first agnostic marker in the history of cancer therapy [15]. Nevertheless, by using an IHC approach or a DNA-based panel, a mismatch-repair deficiency is only observed in a small fraction of tested patients, suggesting that a wider gene analysis may represent a better approach to identify patients with a high number of somatic mutations, related to a high number of neoantigens. Very recently, TMB has been validated in first-line NSCLC setting as positive predictive biomarkers to evaluate the PFS benefit for nivolumab plus ipilimumab treatment. The role of TMB as positive predictive biomarker has also been evaluated in a large retrospective series of cancer patients treated with different immune-checkpoint inhibitors, showing a very promising transversal role as positive predictive biomarker [14]. In addition to positive predictive factors, translational research is looking for biomarkers to identify the subset of patients where immunotherapy seems to stimulate tumor growth, raising the phenomenon known as “hyperprogression” [106]; the innate immunity could be involved in this process [107].

**Figure 2:**
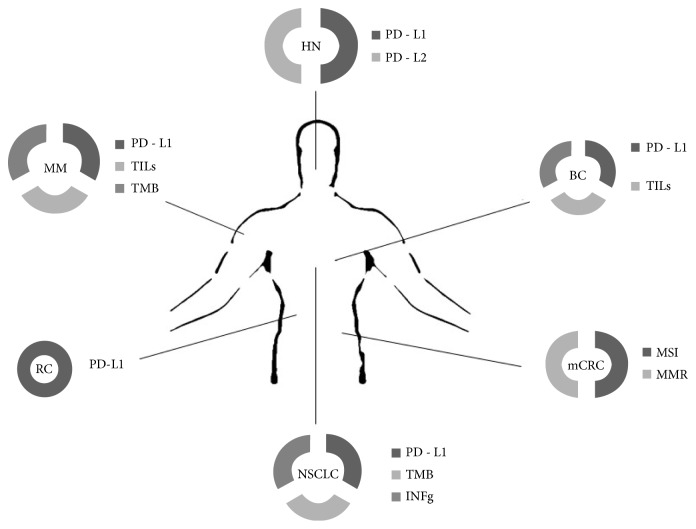
In the figure are reported the principal biomarkers evaluated in clinical trials across different tumor types (MM: melanoma, NSCLC: Non-Small Cell Lung Cancer; mCRC: metastatic Colorectal Cancer; BC: Breast Cancer; HN: Head and Neck Cancer; RC: Renal Cancer).

In conclusion, anticytotoxic CTLA-4, PD-1/PD-L1 checkpoint inhibitors have a relevant clinical value in a large number of solid tumors (Table 1 and Figure 2), becoming a new pillar in cancer therapy but with an efficacy limited by intrinsic or acquired resistance to these agents. In this exciting but very complex context there is the urgent need to overcome the naïve vision of a single biomarker to identify patients that are most likely to respond to ICB therapy. To this end, the integration and simultaneous evaluation of clinical relevant biomarkers, identifying independent population of cancer patients that may experience a clinical benefit, (e.g., PD-L1 and TMB in NSCLC patients) may represent a better solution to translate in everyday clinical practice the results obtained in clinical trials.

## Figures and Tables

**Table 1:** Biomarkers evaluated in relation to immunotherapy regimen in different solid tumors.

Primary Tumor	Immune-checkpoint inhibitor/s	Biomarker	References
Melanoma	Pembrolizumab	PD-L1	[16]
	Pembrolizumab	TILs	[21, 22]
	Nivolumab	TILs	[22]
	Nivolumab+Ipilimumab	PD-L1	[17, 18]
	Ipilimumab	TILs	[20]
	Ipilimumab	TMB	[26, 27]
	Tremelimumab	TMB	[27]

NSCLC	Pembrolizumab	PD-L1	[6, 34–36]
	Pembrolizumab	TMB	[24]
	Pembrolizumab+CT	PD-L1	[37, 38]
	Nivolumab	PD-L1	[39–42]
	Nivolumab	TMB	[14, 39]
	Nivolumab+Ipilimumab	TMB	[14]
	Atezolizumab	PD-L1	[43–45]
	Atezolizumab	T-effector/ IFN-*γ* gene signature	[43]
	Atezolizumab	bTMB	[46]
	Atezolizumab+CT	PD-L1	[47–49]
	Durvalumab	PD-L1	[50, 51]
	Durvalumab+Tremelimumab	PD-L1	[52]
	Avelumab	PD-L1	[53, 54]

CRC	Pembrolizumab	dMMR	[25]
	Nivolumab	dMMR	[55]
	Nivolumab+Ipilimumab	dMMR	[56]

HNSCC	Nivolumab	PD-L1	[57]
	Pembrolizumab	PD-L1	[58–60]
	Pembrolizumab	PD-L2	[60]

RCC	Nivolumab	PD-L1	[61]
	Nivolumab+Ipilimumab	PD-L1	[62]

GGC	Pembrolizumab	PD-L1	[63]
	Nivolumab	PD-L1	[64]

HCC	Nivolumab	PD-L1	[65]

UGC	Pembrolizumab	dMMR	[66]

BC	Pembrolizumab	PD-L1	[67–73]
	Pembrolizumab	TILs	[69, 70, 73]
	Pembrolizumab	TMB	[72]
	Avelumab	PD-L1	[74]
	Atezolizumab	PD-L1	[75, 76]
	Atezolizumab	TILs	[76]
	Durvalumab	TILs	[77]

Abbreviations. NSCLC: Non-Small Cell Lung Cancer; CRC: Colorectal Cancer; HNSCC: Head and Neck Squamous Cell Carcinoma; RCC: Renal Cell Carcinoma; GGC: Gastric and Gastroesophageal Junction Cancer; HCC: Hepatocellular Carcinoma; UGC: Upper Gastrointestinal Cancers; BC: Breast Cancer; CT: chemotherapy; PD-L1: Programmed Death Ligand-1, TILs: Tumor-Infiltrating Immune Cells; TMB: Tumor Mutational Burden; bTMB: Blood Tumor Mutational Burden; dMMR: Deficient Mismatch Repair.
